# Supplementary material for: A nanofabricated, monolithic, path-separated electron interferometer
Source: Sci Rep. 2017 May 10;7:1677. doi: 10.1038/s41598-017-01466-0 (PMC5432008; doi:10.1038/s41598-017-01466-0)
Supplement: Supplementary file 1 — Supplementary Information [file 41598_2017_1466_MOESM1_ESM.pdf]

A nanofabricated, monolithic, path-separated electron interferometer
Supplemetary Information

Akshay Agarwal[1], Chung-Soo Kim[1], Richard Hobbs[1], Dirk van Dyck[2], and Karl K. Berggren[1,*]

[1]Department of Electrical Engineering and Computer Science, Massachusetts Institute of Technology, Cambridge, MA USA
[2]EMAT, University of Antwerp, Groenenborgerlaan 171 2020 Antwerp, Belgium
[*]berggren@mit.edu

1 Fabrication

The fabrication process is outlined in figure S1. We fabricated the two-grating interferometers by gallium FIB milling (FEI Helios Nanolab 600 and 650) of single-crystal silicon (110) cantilevers on tungsten TEM grids (Nanomesh, from Hitachi High-Tech). The gratings were made on one monolithic silicon (110) crystal cantilever with two thicknesses (5 and 40 µm) (Figure S1(a) and inset). We initially placed the cantilever perpendicular to the ion-beam optical axis. The first step was milling of a window (through hole) through the 5/40 µm thick silicon cantilevers using 30 kV gallium ion beam, as seen in figure S1(b). This window defined the lateral extent of the grating. It also acted as a milling stopper, and helped to reduce material re-deposition and secondary sputtering in subsequent steps. We then placed the cantilever along the direction of the optical axis and deposited two 3 µm thick platinum layers to define the gratings, and protect them from ion-beam damage. The deposited platinum layers are seen in figure S1(c). Next we milled the unprotected silicon at 30 kV and 21 nA beam current. This step at large current and energy milled most of the silicon between the gratings, as shown in figure S1(d) It was important here to leave substantial (~ 500 nm) thickness at each grating to allow for some beam focusing errors and resulting damage in the non-milled area. The gratings were polished at successively lower currents (down to 50 pA) and energies (down to 2 kV). This step thinned down the gratings to less than 50 nm and removed most of the amorphous layer formed from ion-beam damage. The final polishing was done at a slight tilt (up to 5°in either direction) to improve the uniformity of thickness each grating. We restricted the lateral dimensions of each grating to be 10 µm by 10 µm to avoid bending of the membranes. The final grating structure is seen in figure S1(e).

2 Mechanism of electron diffraction from multiple crystal gratings

We used electron diffraction from the two-grating structure to test the mutual alignment of the gratings as described in the paper. As shown in figure S2(a), the two-grating diffraction pattern from the 20 µm structure with a nearly parallel (convergence semi-angle $\alpha = 0.2$ mrad) beam is identical to that from the 2.5 µm-gap-structure.

The convergent beam diffraction pattern (DP) in figure S2(b) can be considered as a regular silicon (110) DP on which another DP with the same symmetry but large demagnification is superimposed. The demagnification of the smaller DP in S2(b) is 0.15. Any attempt to explain this DP as a regular Moiré pattern runs into difficulties because a ~ 15 % difference in lattice constant between the two gratings (induced by stress or thermal expansion) is unlikely. Further, the disappearance of the demagnified pattern for a nearly parallel beam also cannot be explained using this mechanism. This indicates that the DP is caused by the

convergence of the beam.

2.1 Convergent beam diffraction from a single-grating structure

A convergent beam can be thought of as a set of plane waves from all the directions within the cone. When this beam is incident on a grating such that it is focused some distance below the grating, each incident plane is diffracted at the grating and generates plane waves that leave the crystal in the directions given by Braggs law. If the incident beam is inclined, the diffracted beams are inclined at the same angle. Thus each diffracted beam will leave the grating as a convergent cone but with the symmetry axis along the Bragg direction and will also converge to a small spot in the same focus plane below the sample as the incident beam, which we called the first crossover plane CP1 in figure 3 (b). The position of CP1 only depends on the setting of the condenser lens and not the position of the object. Crucially, the distance between the object and CP1 determines the gap between the spots in the CP1; the smaller this distance, the smaller the gap between the spots.

2.2 Convergent beam diffraction from a multi-grating structure

With two (or more) gratings, the CP1 of the convergent beam remains the same, but now, due to the different vertical positions of the gratings along the optical axis, the distance between the focused diffracted spots from each grating is different. Therefore at CP1 we will see multiple superimposed DPs with the same symmetry but different magnifications. To image these focused beams, we need to tune the intermediate lens current to move below the back focal plane (where the convergent beams result in disks as seen in figure 3 (c) to the 'second crossover plane' CP 2, as shown in figure 3(b). At this plane, we image the CP 1 and hence see the DP with multiple, superimposed magnifications. By taking all possible orders of diffraction into account, rather than just the first as in figure 3 (b), we can build up the experimental DP. This imaging mode bears some resemblance to conventional convergent beam electron diffraction (CBED), with the important difference that the beam crossover in CBED is focused inside the sample, whereas here it is focused several microns below the sample.

To verify this explanation of the observed diffraction pattern we performed the following experiments:

1. We first verified that there is significant horizontal displacement between the zero and first order diffracted beams at CP 2 from a single layer of silicon with a convergent beam. We fabricated a single grating sample and recording the diffraction pattern from it at CP 2 and different stage heights in the TEM. As can be seen from figure S3(a), this reciprocal space distance between the primary and first order diffracted spots decreases linearly with stage height. The red and blue data points depict two

different beam semi-convergence angles $\alpha_1 = 5$ mrad and $\alpha_2 = 10$ mrad . This change in distance did not occur when imaging in the back focal plane with a parallel beam. This observation confirmed that the source of the change in distance was the convergence of the beam. As described earlier, the two-grating diffraction pattern can be thought of as a superposition of the patterns from a single layer at two heights separated by the inter-grating gap D (with additional spots from double diffraction), and this experiment indicated that the spots from the two gratings were horizontally displaced due to the convergent of the beam. Also, by extrapolating the best fit curves, we calculated that the convergent beams were focused $\sim$ 14 and 26 µm below the eucentric plane for α_1 and α_2 respectively. This plane was the position of CP 1 in this experiment.

2. We next placed the 2.5 µm gap two-grating sample in the two-beam condition by tilting the TEM sample holder, so that we could focus on just one diffraction order($\mathbf{g}_{\text{net}} = (0\bar{2}2)$) besides the primary beam. We then varied the beam convergence angle (by changing the beam spot size) and recorded the DP at CP 2. Figure S3(b) and (c) show the recorded DP for $\alpha =$ of 10 and 1 mrad. The gap between the two $(0\bar{2}2)$ spots arising from the beams $\Psi_{\mathbf{0g}}$ and $\Psi_{\mathbf{g0}}$ (with $\mathbf{g} = (0\bar{2}2)$)) reduced at a lower convergence angle. This experiment verified that the gap between the diffracted spots from the two gratings could be tuned by changing α, as expected from the proposed mechanism.

3 Beam diameter and convergence angles for separation

To determine the optimal beam diameter for simulations, we performed preliminary experiments with beam diameters ranging from 60-300 nm and α between 0.5 and 5 mrad. On the JEOL 2010F, beam diameters between 60-200 nm with α less than 4 mrad required the use of a very small (10 μm) condenser aperture which severely limited the intensity of the beams. The reduced beam intensity increased the exposure time required to record interference fringes. The increased exposure time resulted in poor fringe contrast due to stage vibrations. Thus, we chose a beam diameter greater than 200 nm and a relatively large α of 4 mrad.

Figure S4 depicts in red the range of beam diameters (on the first grating) and convergence angles that prevents the beams with $\mathbf{g}_{\text{net}} = (000)$ and $\mathbf{g}_{\text{net}} = (1\bar{1}1)$ from overlapping with each other at the second grating and the interference plane. The values in blue violate either one or both of these requirements. These values were obtained from the GSM simulations outlined in the paper, for a 200 kV beam and 20 µm gap gratings. The interferometry results in the paper are for the beam parameters indicated by the yellow point (diameter 240 nm, $\alpha = 4$ mrad).

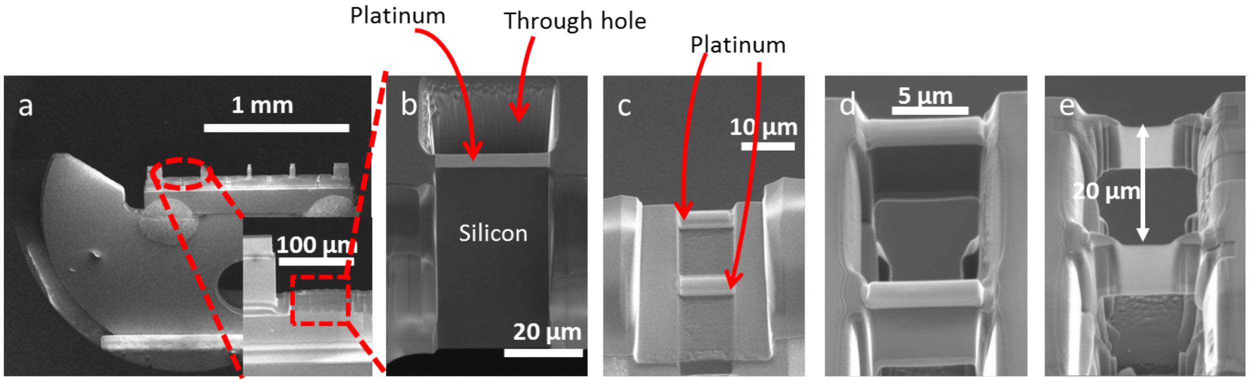

Figure S1: FIB fabrication of the monolithic two-grating interferometer. (a) We started with single-crystal silicon cantilevers on a regular TEM grid. Inset shows a zoom of one of the cantilevers. (b) We deposited platinum on the top surface and milled a window through the cantilever to define the lateral area of the gratings (c) The grid was rotated by 90°and two 3 µm- thick platinum layers were deposited on the silicon to protect the gratings (d) The silicon between the platinum layers was milled at high ion-beam energy (30 kV) and current (21 pA) (e) The two gratings were thinned down and polished to a final thickness of ~ 45 nm by lowering the ion-beam energy down to 2 kV.

4 Misalignment of 20 µm interferometer

Figure S5 shows three TEM images of the $\Psi_{0\mathbf{g}}$ and $\Psi_{\mathbf{g}\bar{\mathbf{g}}}$ ($\mathbf{g} = (1\bar{1}1)$)) beams near the interference plane. Figure S5 (a) was taken 1 µm above the interference plane, Figure S5 (b) at the interference plane and Figure S5 (c) 1 µm below the interference plane. The beams were focused for ease of viewing and quantification of misalignment. As seen in figure S5 (b), in the interference plane, the focused beams are separated by 4.5 nm in a direction perpendicular to the direction of beam overlap. This perpendicular separation suggests that the two gratings are rotationally misaligned by $\theta_{\text{misalign}} = 4.5\,\text{nm}/20\,\mu\text{m} \approx 220\,\mu\text{rad}$.

Note that the interference fringes reported in the paper were obtained by defocusing the $\Psi_{0\mathbf{g}}$ and $\Psi_{\mathbf{g}\bar{\mathbf{g}}}$ beams from figure S5, so that they overlapped.

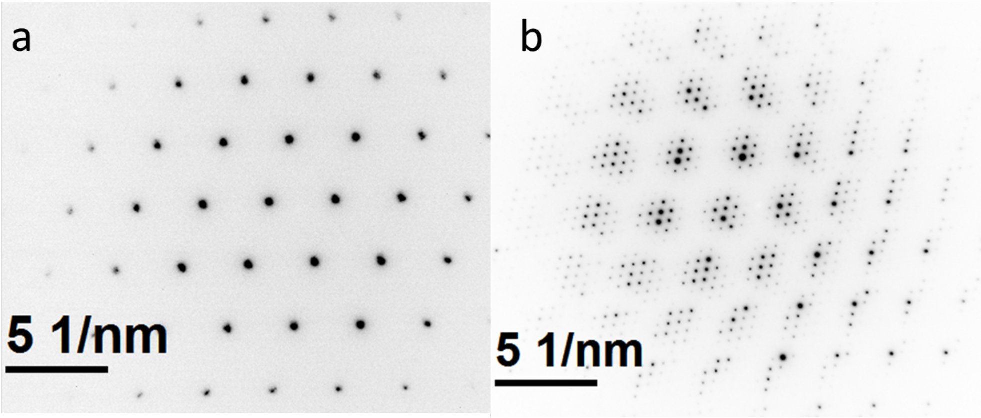

Figure S2: Electron diffraction from 20 µm-gap-structure. (a) With a nearly parallel beam, we obtained a diffraction identical to single layer silicon. (b) With a convergent beam we obtained multiple closely-spaced spots just as for the 2.5 µm-gap-structure.

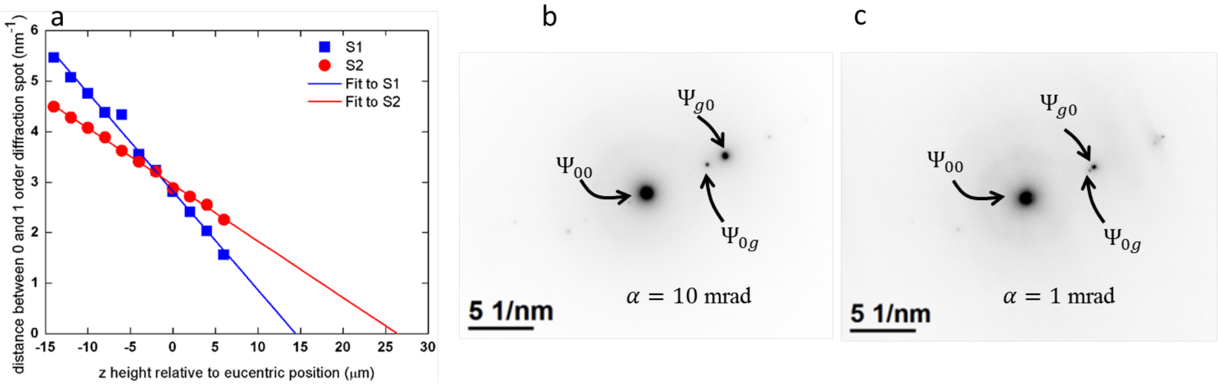

Figure S3: Verifying the role of beam convergence in creating multiple diffraction spots. (a) Reciprocal space distance between primary and first order ($\mathbf{g} = (1\bar{1}1)$)) diffracted spots from a single layer of silicon in CP2, as a function of the stage height z relative to the eucentric plane, for convergence angles $\alpha_1 = 5$ mrad (red circles) and $\alpha_2 = 10$ mrad (blue squares). The solid lines are linear fits to the data points. The distance between the spots changed with the stage height for convergent beams. (b) At a large convergence semi-angle ($\alpha = 10$ mrad), the diffraction spots from the $\Psi_{\mathbf{0g}}$ and $\Psi_{\mathbf{g0}}$ beams ($\mathbf{g} = (0\bar{2}2)$)) at CP2 were distinct. (c) At a smaller convergence semi-angle ($\alpha = 1$ mrad), the two diffraction spots were much closer to each other.

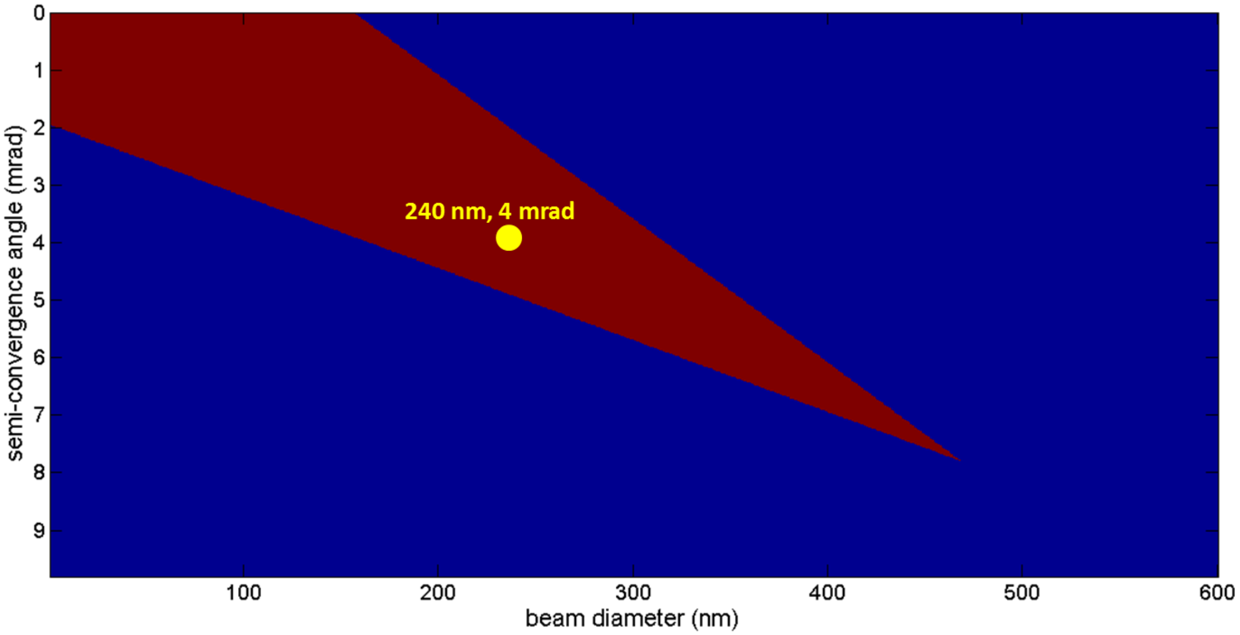

Figure S4: Allowed values of beam semi-convergence angle and diameter at the first grating. The parameters for which results are reported in the paper are indicated by the yellow point.

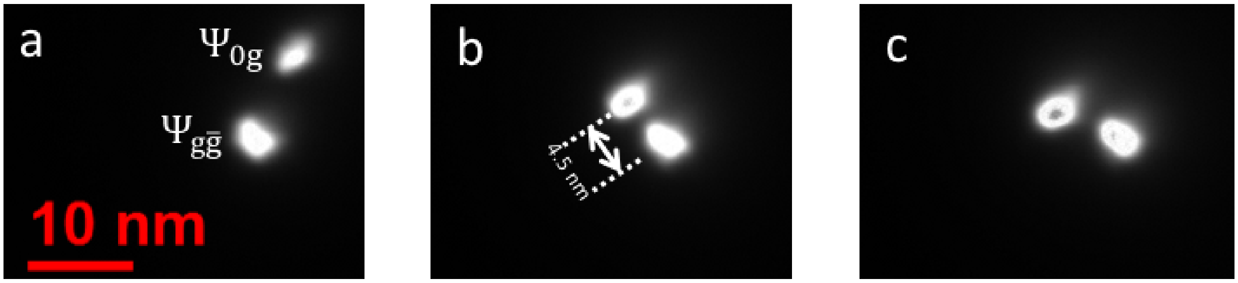

Figure S5: $\Psi_{\mathbf{0g}}$ and $\Psi_{\mathbf{g\bar{g}}}$ beams (focused for ease of viewing) at (a) z=-19 µm (b) z=-20 µm (*i.e.*, the interference plane and (c) z=-21 µm below the second grating.
